# Update on Nonhuman Primate Models of Brain Disease and Related Research Tools

**DOI:** 10.3390/biomedicines11092516

**Published:** 2023-09-12

**Authors:** Nan Qiao, Lizhen Ma, Yi Zhang, Lifeng Wang

**Affiliations:** 1 School of Life Sciences, Hebei University, 180 Wusi Dong Lu, Baoding 071002, China; nqiao23888@163.com; 2 Beijing Institute of Radiation Medicine, 27 Taiping Road, Beijing 100850, China; malizhen0906487@sina.com

**Keywords:** NHPs, brain disease models, EEG, MRI, optogenetics, neural circuits

## Abstract

The aging of the population is an increasingly serious issue, and many age-related illnesses are on the rise. These illnesses pose a serious threat to the health and safety of elderly individuals and create a serious economic and social burden. Despite substantial research into the pathogenesis of these diseases, their etiology and pathogenesis remain unclear. In recent decades, rodent models have been used in attempts to elucidate these disorders, but such models fail to simulate the full range of symptoms. Nonhuman primates (NHPs) are the most ideal neuroscientific models for studying the human brain and are more functionally similar to humans because of their high genetic similarities and phenotypic characteristics in comparison with humans. Here, we review the literature examining typical NHP brain disease models, focusing on NHP models of common diseases such as dementia, Parkinson’s disease, and epilepsy. We also explore the application of electroencephalography (EEG), magnetic resonance imaging (MRI), and optogenetic study methods on NHPs and neural circuits associated with cognitive impairment.

## 1. Introduction

In recent years, as the world population has aged, the prevalence and incidence of cognition-related diseases (dementia, Parkinson’s disease, epilepsy, etc.) have increased, which places great stress on society and families. Some of these diseases have high mortality rates, and their etiology and pathogenesis are unclear, while their main manifestations include deficits in learning and memory function, language, visuospatial function, executive function, personality, and attention, among others [1,2,3,4].

Alzheimer’s disease (AD) is the most common type of dementia, accounting for 60–70% of cases [5], and has become one of the major fatal diseases in the elderly population; despite research efforts, there is currently no effective treatment for this condition. At present, there are approximately 50 million AD patients in the world. With the aging of the world population, it is estimated that the number of Alzheimer’s disease patients will almost triple to 140 million in 2050 [6]. Patients with AD typically exhibit amnestic aphasia in the early stages, and as the disease progresses, it is mainly characterized by loss of progressive memory and acquired knowledge until daily living abilities are completely lost [7].

Parkinson’s disease (PD) has a prevalence of 2–3% among people aged 65 years and older, and it is the second most common neurodegenerative disease in the world [8]. With the rapid aging of the population, the number of PD patients is increasing year by year, which places huge medical and economic burdens on society and families. The PD prevalence in people over 65 years old in China is approximately 1.7%. The main pathological change in PD patients is the degeneration and death of dopamine neurons in the substantia nigra of the midbrain, which leads to a significant decrease in the DA content of the striatum [9]. The exact etiology of this pathological change remains unclear, and genetic factors, environmental factors, aging, and oxidative stress may be involved in the degenerative death of dopaminergic neurons in PD.

Epilepsy is a common neurological disorder that affects approximately 1% of the world’s population; it is characterized by long-term and unpredictable seizures caused by abnormal neuronal electrical activity. Epilepsy is caused by the sudden abnormal discharge of brain neurons, which leads to temporary brain dysfunction [10]. According to the latest epidemiological data from the China Association Against Epilepsy, the overall prevalence rate of epilepsy in China is 7.0‰, and the annual incidence rate is 28.8/1 × 10^5^. Based on this, it is estimated that there are approximately nine million epilepsy patients in China. Meanwhile, there are approximately 400,000 new epilepsy patients added each year, making epilepsy the second most common disease in neurology after headaches in China.

With the aging of the world population, it is particularly important to select appropriate, safe, effective, and ideal experimental animal models for the study of senile diseases. Based on the principle of selecting similarity in experimental animals, the advantages of nonhuman primate models are becoming increasingly prominent.

Due to the connection between shared sequences and the evolution of environmental forces, NHPs have many anatomical and functional similarities to humans. Compared to rodents, NHPs have cortical folding, segmentation, and expansion that are more similar to humans, and these features increase the complexity of their anatomy, cognition, and behavior, making it difficult to find perfectly consistent models of many human neurological diseases in rodents. Macaques are the most studied NHPs [11,12]. The establishment of an NHP brain disease model is crucial to unravel the mechanisms of human diseases and to develop more in-depth therapeutic strategies.

**Figure 1:**
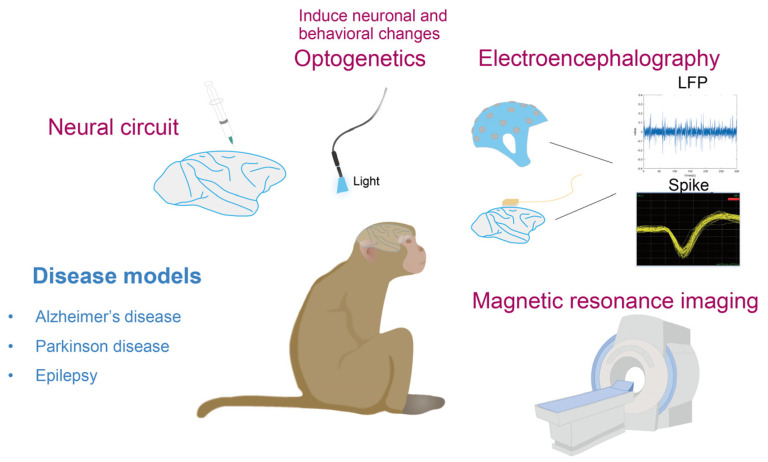
A summary of disease models, brain function and imaging techniques in NHPs.

The emergence and development of techniques such as electroencephalography (EEG), magnetic resonance imaging, and optogenetics have revealed new horizons for neuroscience research, and these methods have resulted in some breakthroughs in rodents. Therefore, understanding the application of these research methods in NHPs is of great significance for future disease research. In this review, we retrieved the keywords AD, PD, epilepsy, EEG, MRI, or virus vectors in the PubMed database and discussed studies on NHP models of cognition-related diseases; the application of EEG, magnetic resonance imaging, optogenetics, and other research methods; and research on disease-related neural circuits (see Figure 1).

Nonhuman primates are ideal neuroscience models for studying the brain. In this review, we summarize three models of common diseases: (1) Alzheimer’s disease; (2) Parkinson’s disease; and (3) epilepsy. In addition, we list studies using EEG, magnetic resonance imaging, and optogenetics in NHPs. The study of neural circuits can fully reveal how the brain performs complex functions; recombinant viruses have become the most commonly used tool for studying neural circuits, and we summarize the common viral vectors applied to neural circuits and discuss their advantages and disadvantages while listing the neural circuits associated with cognitive impairment.

## 2. Models of Human Cognitive-Related Brain Disease in NHPs

Of all animal research models, NHPs are the closest to humans in terms of physiology, anatomical structure, and immunity. Long-term practice has demonstrated that NHPs provide appropriate models of human disease and have natural advantages in this function. NHPs are the most suitable for exploring human disease mechanisms and new treatment modalities. Therefore, NHPs are a highly reliable experimental model species in which to study the etiology, pathogenesis, and potential treatment of brain diseases.

### 2.1. AD Models

The cognitive impairment of AD patients mainly manifests as learning and memory impairment, language impairment, visuospatial impairment, and executive dysfunction, among other deficits. The main pathological manifestations of AD are extracellular β-amyloid (Aβ) deposition, tau protein abnormalities, intracellular neurofibrillary tangles, and neuronal loss [13]. The value of using NHPs as a disease model is undeniable; for example, macaques have greater than 98% homology to human tau protein and 100% homology to human Aβ sequences [14].

The AD models that exist at present include natural aging models, transgenic animal models, models constructed by physical or chemical methods, and models constructed by a combination of methods. However, these models are mostly based on C57BL6/J mice, while there have been few studies on macaques. Yang et al. used macaques as research subjects. Juvenile male macaques were chronically fed 3% methanol for 6 months, and a variable spatial delayed-response task was administered. The results showed a persistent decline in the memory capacity of these macaques, which was consistent with the abnormal increase in tau protein phosphorylation of T181 and S396 residues in cerebrospinal fluid during methanol feeding, as well as an increase in tau protein phosphorylation aggregates and amyloid plaques. The phenomena are present in four regions of postmortem brains: the prefrontal cortex (PFC), the parietal cortex, the temporal cortex, and the hippocampus. Subsequently, the researchers fed macaques methanol for 6 months and found persistent changes in phosphorylated tau protein in the brain after consuming methanol, suggesting that methanol feeding leads to long-term pathological changes related to the development of AD in macaques [15]. This result provided a new perspective and means for the prevention and treatment of AD and was also the first rhesus monkey model with the core symptoms of AD. Beckman D et al. [16] injected a total of 100 μg Aβ oligomers (AβOs) into one lateral ventricle of rhesus monkeys (divided into 8 injections, given over the course of 24 days). After one week of modeling, the pathological examination found that the rhesus monkeys showed synaptic damage and neuroinflammation, but there were no Aβ- or tau-related pathological changes and no decline in cognitive function. The following year, Beckman et al. [17] injected adeno-associated viruses expressing double mutation tau—(AAVP301L/S320F)—twice into the left hemisphere of the entorhinal cortex (ERC) of rhesus monkeys, and the right hemisphere ERC was injected with AAV–green fluorescent protein as a control. After three months, all animals showed abnormal tau phosphorylation and neurofibrillary tangles in the brain, similar in distribution to those of AD patients. In addition, the model also showed similar human 4R tau and 3R tau to those of AD patients, but this model did not show Aβ-related pathological changes or cognitive decline. Although these studies did not simulate the full range of symptoms of AD, they made a great contribution to the establishment of NHP AD models, and the researchers also raised the new question of whether all symptoms of AD could be elicited by multiple injections or by increasing the induction time, which also needs to be further investigated.

### 2.2. PD Models

The core symptoms of PD are bradykinesia and clinical manifestations such as static tremor, myotonia, and abnormal posture [8]. Typical pathological features include massive loss of dopaminergic neurons in the substantia nigra pars compacta of the midbrain [18,19] and the presence of Lewy bodies (LBs) formed by aggregation of phosphorylated α-synuclein in the associated brain regions [18].

In 1959, Senoh and Witkop [20] first isolated 6-OHDA, a hydroxylated derivative of catecholamines, and later simulated PD-like physiological and pathological changes in primates through 6-OHDA. Although the 6-OHDA-induced PD animal model features the selective destruction of nigrostriatal dopaminergic neurons with no effect on other neurons, the most dominant motor deficit appeared in the form of lateral rotation, which does not closely mimic the common clinical signs of PD and the typical pathological marker LB of PD [21]. In the 1980s, 1-methyl-4-phenyl-1,2,3,6-tetrahydropyridine (MPTP), an effective dopaminergic neurotoxin, was first used to induce the symptoms of PD in macaque monkeys [22], but whether LB will be induced is controversial. In the early 21st century, it was reported that misfolded α-synuclein might be responsible for triggering PD [23,24], but there have been relatively few studies on this topic in NHPs, and no behavioral changes have been reported.

Li et al. [25] combined the known PD risk genes and their possible pathogenic mechanisms, used AAV-mediated CRISPR/Cas9 technology to directly edit PINK1 and DJ-1 genes in the substantial nigra region of adult macaques, and successfully built the first gene-edited adult macaque PD model. This novel aetiological macaque model provides an irreplaceable research platform for aetiological exploration, early marker discovery, and the development of effective intervention and treatment strategies for PD. However, it is unclear to what extent the model truly simulates the development of early-onset familial PD.

### 2.3. Epilepsy Models

In approximately one-third of epilepsy patients, these seizures cannot be controlled through drugs; this condition is known as refractory epilepsy. Patients with refractory epilepsy have significantly greater mortality, physical trauma, and risk of psychological disorders than the overall population of epilepsy patients [26]. The clinical manifestations of epilepsy are complex and varied, encompassing paroxysmal motor, sensory, autonomic nerve, consciousness, and mental disorders. The etiology of epilepsy is diverse, and its pathogenesis is very complicated.

Systemic convulsants are often used in the development of models of temporal lobe epilepsy in primates. However, this usually leads to diffuse brain damage that affects multiple lesions outside the limbic system. Perez-Mendes et al. [27] induced an epilepsy model in marmosets by intraperitoneal injection of 250 mg/kg pilocarpine, which produced bilateral hippocampal sclerosis and granular cell dispersion. Hong et al. [28] repeatedly injected rhesus monkeys with subthreshold doses of Coriaria lactone and found that it could cause partial seizures and eventually lead to secondarily generalized tonic-clonic seizures without any sign of hippocampal sclerosis. However, these two models produced convulsive seizures during chronic epilepsy, which may result in extensive neuronal damage in the brain. Chen et al. [29] established a rhesus monkey model of chronic temporal lobe epilepsy by injecting kainic acid (KA) into the amygdala, and Chi et al. [30] established a similar model by injecting KA into the hippocampus. Neither model showed any obvious signs of clonus or convulsions during chronic status epilepticus.

## 3. Current Status of Research and Application of Brain Function and Imaging Techniques in NHP Models

In cognitive studies of humans and NHPs, the cerebral cortex is recognized to consist of anatomically and functionally distinct cortical regions. However, these cortical regions complete tasks independently in use or rapidly and selectively in a flexible way to achieve different effects. Neural network theory proposes two basic characteristics of brain function, namely, functional separation and functional integration [31,32]. For optimal network and cognitive function, the balance between functional integration and functional separation is crucial [31]. In the field of brain function research, NHPs have provided important value for humans to explore brain functions and brain diseases, and EEG and MRI are the main techniques used to study brain function. In recent years, the development of optogenetic technology has also opened a new perspective for the study of brain function in macaque monkeys.

### 3.1. Classification of Neuroelectrical Signals

An EEG signal is the spontaneous, rhythmic electrical activity of brain cell clusters recorded by electrodes. EEG is an effective method for examining functional changes in the brain and is highly sensitive to changes in the neural activity of the brain at the millisecond level [33]. According to the physical depth at which measurements are taken, EEG can be subdivided into scalp EEG, which consists of signals recorded at the scalp surface; electrocorticography (ECoG), which is recorded at the surface of the cerebral cortex/dura mater; and local field potentials (LFPs) and spikes, which are recorded from the interior of the brain.

#### 3.1.1. Scalp EEG

Scalp EEG represents the electrical activity of a large brain region recorded from the scalp surface. Because of its noninvasive nature and portable, easy-to-use apparatus, it is safe for patients and is increasingly used in various neurodegenerative and neuropsychiatric diseases, such as AD, schizophrenia, depression, Huntington’s disease, PD, multiple sclerosis, and brain injury [34,35]. For example, Chen et al. [36] injected KA 3–4 times a week into the unilateral hippocampus of five cynomolgus monkeys to induce repetitive acute seizures. During the process, EEG was used to monitor KA-induced acute seizures and subsequent spontaneous recurrent epileptiform discharges (SREDs). However, EEG is limited in the spatial and temporal domains and has a lower signal-to-noise ratio than ECoG and LFP [37]. It is undeniable that EEG can be directly applied to humans and can be used to discover a large amount of physiological and pathological information. However, ECoG and LFP are more advantageous for physical, chemical, and pharmacological intervention studies.

#### 3.1.2. ECoG

ECoG is a technique that uses subdural electrodes to record neural activity on the cortical surface, allowing simultaneous recording of neural activity from several of these electrodes without penetrating the cortical tissue. ECoG has higher spatial resolution (i.e., 0.1 mm vs. cm), wider bandwidth (i.e., 0–500 Hz vs. 0–40 Hz), higher amplitude (i.e., maximum 50–100 µV vs. 10–20 µV) [38,39], and a much higher signal-to-noise ratio than noninvasive techniques such as EEG [40]. Because ECoG can cover a wide range of cortical regions, it has recently become an important tool for studying functional interactions between cortical areas for various sensory, motor, and cognitive functions [41,42]. Cauchoix et al. [43] recorded multichannel subdural ECoG signals in the middle area of the ventral stream of the visual cortex (V4/PIT) when a rhesus monkey was performing the animal/nonanimal classification task. The study found that the middle region of the ventral flow of the visual cortex (V4/PIT) correlated with visual selection and classification and confirmed that macaques and humans exhibit similar correct and false response patterns in rapid object classification. The application of ECoG to record information carried on different time scales, such as γ and θ oscillations, has also attracted increasing attention [42,44,45].

#### 3.1.3. LFPs

LFPs are the low-frequency component of the electrophysiological signal recorded invasively in the brain, which can capture neural oscillations at approximately 1–300 Hz. LFP recording has become an important technique for neurophysiological recordings in the cerebral cortex. Because LFP recording is suitable for simultaneous measurements from different regions, it enables the study of neural interactions in different brain regions. LFP can capture the traditional single-unit activity (SUA) to record otherwise unmeasurable synaptic processes [46].

Spike signals and LFP signals can be separated from the electrophysiological signal recorded by the implanted electrode. Spikes primarily represent a series of action potentials emitted by a single neuron and are the output from neurons within a radius of approximately 200 μm around the tip of the microelectrode [47]. LFPs are thought to come mainly from dendritic activity, reflecting the synaptic activity near the electrode, with a spatial range from hundreds of microns to several millimeters [42,48]. Therefore, analysis of simultaneous LFP and spike recordings can provide important information on connections between individual neurons and local network activity [49]. Confais et al. [50] simultaneously recorded LFP signals and spike signals from multiple electrodes in the macaque motor cortex during a visuomotor latency center-outwards extension task and demonstrated that there is no internal relationship between the discharge frequency of a single neuron in the rhesus monkey motor cortex and the amplitude of β oscillation. Cohen et al. [51] measured the spike signals in two male rhesus monkeys under auditory stimulation during the detect-type task and detect-location tasks and found that ventrolateral prefrontal cortex neurons were reliably modulated during the detect-type task but not during the detect-location task. Furthermore, the degree of modulation during the nonspatial task correlated positively with the monkeys’ behavioral performance.

One sign of LFPs is that the oscillations of various frequencies are modulated between different times and different brain structures. LFP oscillations at different frequencies are related to different sensory and cognitive processes and information transmission between brain regions [52]. Yan et al. [53] found that spike pulse coherence between the frontal eye field (FEF) and V4 was enhanced in the theta band (4–8 Hz) but suppressed in the alpha band (8–13 Hz) when male rhesus monkeys performed a free-gaze search. Doucet et al. [54] measured the signals of hippocampal LFPs and spikes when two male rhesus monkeys performed different tasks with unconstrained eye movements; the team found that signals linked to eyekicks reached the hippocampus, producing the synchronization of delta/theta LFPs without general activation of local neurons. Moreover, the authors found that some visual inputs co-occurring with saccades produced LFP synchronization in the alpha/beta bands and enhanced neuronal firing. They speculated that the signals associated with saccades might enact sensory input-dependent plasticity and, therefore, memory formation in the NHP hippocampus.

#### 3.1.4. The Correlation of EEG Was Found in Different Brain Regions

EEG has also been applied to study the correlations of different parts of the brain. Some studies have shown that both action selection and spatial working memory are associated with the frontoparietal network of the primate cerebral cortex, particularly with direct interactions between the frontal and parietal lobes [55], but how these spatially distant cortical circuits coordinate their activities into a unified functional network is still poorly understood.

Although some researchers have made great contributions regarding the interaction between the frontal and parietal lobes, the neural mechanism underlying the control of the frontoparietal network remains unresolved. Dotson et al. [56] simultaneously recorded neuronal activity from multiple electrodes on the prefrontal and posterior parietal cortex as female rhesus monkeys performed an eye-movement delayed matching task and estimated the incidence, size, and relative phase angle of time-related activities through cross-correlation analysis. They found that the short-range and long-term correlations showed significant task-dependent changes in intensity and relative phase, confirming that cognitive events are accompanied by strong changes in the temporal coordination pattern of the frontoparietal network. Martínez-Vázquez et al. [55] recorded the neuronal activity of multiple electrodes in the extended parietal area and dorsal premotor area in macaque monkeys. The study found that the two interacted functionally to achieve cognitive control during target-oriented behavior, especially the frontal-parietal interaction that occurred during the retrieval of moving target information from spatial working memory, and found that β/γ activity dominated the influence of the parietal lobe on the frontal lobe, while low-frequency activity dominated the influence of the frontal lobe on the parietal lobe.

Other researchers have proposed that the coherent oscillation in neuronal activity constitutes a hypothetical dynamic mechanism for regulating the interaction between different brain regions [53,57]. Simultaneous recordings from multiple regions in the cerebral cortex of macaque monkeys have shown that continuous high-frequency (β and γ) oscillations are associated with multiple cognitive functions [58,59,60]; however, further analysis is still needed to corroborate this correlation. Whether such oscillations are also present in activity between distant cortical circuits during other cognitive functions must also be further explored.

### 3.2. MRI: A Peek into the Internal Structures of the Brain

MRI is a technique that uses the phenomenon of magnetic resonance to obtain electromagnetic signals from the human body and reconstruct body information, which plays a crucial role in neuroscience research involving NHPs. Before the appearance of MRI, neurophysiologists relied on postmortem histological evaluation of brain sections under a microscope to identify target areas by recording or injection. The emergence of MRI provided a new perspective to further explore the internal structure of the brain and the pathogenesis of brain diseases. Recently, the combined application of MRI and other techniques has greatly improved the accuracy and efficiency of neurophysiological experiments in NHPs [61], offering the opportunity to gain insight into the structure and function of the human brain. MRI can be subdivided into structural magnetic resonance imaging (sMRI) and functional magnetic resonance imaging (fMRI) according to its principles.

#### 3.2.1. Application of Structural Magnetic Resonance Imaging (sMRI) in NHPs

sMRI uses the nuclei of hydrogen atoms arranged in a certain sequence under the action of a magnetic field; in this imaging modality, the changes in some structures can be clearly observed. In NHPs, sMRI can be combined with invasive experimental conditions that cannot be achieved in human research, such as drug intervention, histological research, high-density electrophysiological recording, and injection of viral vectors for optogenetic manipulation or calcium imaging; thus, sMRI provides a unique way to understand the human brain and its pathology [62]. sMRI has also been applied to the study of brain cognitive function and related diseases, such as AD and PD.

Pelekanos et al. [63] observed functional connectivity and white matter structure changes in the cortex and thalamic cortex in rhesus monkeys performing visuospatial discrimination (reward-guided learning) by MRI. Changes in functional connectivity were found in the orbitofrontal lobe, ventromedial prefrontal lobe, inferotemporal lobe, entorhinal lobe, posterior splenic lobe, anterior cingulate cortices, hypothalamic subfasciculus complex, and dorsal and medial thalamus. These changes in the functional connections between the cortical and thalamic cortex are accompanied by changes in the associated white matter structures in the hooked bundle, fornix, and ventral side of the prefrontal lobe. Subsequently, the authors severed the fornix of well-trained macaques and found that their ability to learn new visuospatial discrimination was impaired, as were the changes in the functional connectivity features and white matter structure in the ventral prefrontal tracts. Studies have shown that different communications transmitted between the cortex and thalamic cortical circuits play an important role in learning new visuospatial connections and making reward-guided decisions. MRI has also been used to observe structural brain changes to track cognition-related disease processes [64].

#### 3.2.2. Application of Functional Magnetic Resonance Imaging (fMRI) in NHPs

In 1990, Ogawa et al. [65] obtained blood oxygen level-dependent fMRI (BOLD-fMRI) based on the principle that an increase in the content of oxygenated hemoglobin (HbO_2_) in the functionally active area of the brain leads to enhancement of the magnetic resonance signal. BOLD-fMRI imaging has the advantages of noninvasiveness, strong repeatability, high spatial and temporal resolution, and easy localization of signals. fMRI has developed very rapidly in the field of functional neurological studies of the brain and has been widely used to examine the functioning of brain networks behind various cognitive, emotional, and motor functions in NHPs [66]. Greyson et al. [67] examined the effect of amygdala inactivation on the whole brain tissue of rhesus monkeys using resting fMRI and found that amygdala inactivation destroyed the communication between the cortical amygdala and the network connection across multiple functional brain systems. Dean et al. [68] used fMRI to find that the posterior cingulate gyrus neurons of macaques were involved in visual orientation and attention.

In recent years, the combined application of MRI, electrophysiology, optogenetics, and calcium imaging has enhanced the effectiveness of research on brain function, and researchers can specifically regulate or intervene in local neuronal activity. The combination of electrical stimulation and functional magnetic resonance imaging (es-fMRI) has become a powerful technique for detecting effective connections between local cortical or subcortical stimulation sites and distal brain regions in humans [69,70]. Some studies have combined inositol injection with fMRI of rhesus monkeys [71] to test cross-brain regional coupling by linking neural activity with behavioral causality. Hirabayashi et al. [72] revealed bidirectional network changes and multifaceted behavioral impairments by combining fMRI and chemical genetic silencing network manipulation of sensory activation in macaques.

### 3.3. Brain Function Regulation of NHPs Based on Photogenetic Technology

Optogenetics is the millisecond resolution control of neural activity using genetically encoded light-gated ion channels, or opsin [73], the principle of which is to pump protons out of the cell or anions/cations into the cell through light-activated proteins with cell-type specific expression to activate (or inhibit) certain types of neurons. To date, most optogenetic studies have been performed in rodents and have reported light-induced neuronal and behavioral changes, as well as clinical therapies [74,75]. The clinical application of optogenetic techniques also needs to be fully demonstrated in NHPs.

In 2009, Han et al. [76] successfully expressed opsin ChR2 in the excitatory neurons of the PFC of macaque monkeys for the first time, achieving light-controlled regulation of NHP neurons in vivo, and preliminary studies demonstrated the feasibility of ChR2 expression and behavioral effects in primates. Then, in 2011, another inhibitory opsin, archaerhodopsin (ArchT), was successfully expressed. Since 2012, researchers have found that optogenetic stimulation of different cortical regions can affect eye movement and influence decision-making in macaque monkeys. Cavanaugh et al. [77] expressed ArchT in the superior colliculus (SC) and found that inhibition of SC neurons by optical stimulation resulted in saccadic eye movement defects. Ohayon et al. [78] found that stimulating the frontal eye field (FEF) by optogenetics alone could not effectively induce saccade behavior, but when this technique was combined with conventional microcurrent stimulation, the possibility of causing rapid saccade eye movements increased significantly. Studies have shown that stimulation with the optogenetic construct ChR2 in the experiment produced subthreshold activity that contributed to movement initiation. However, in most cases, subthreshold activity was not enough to elicit a motor response. Inoue et al. [79] expressed ChR2 in neurons projecting from the FEF to the SC and found that optogenetic stimulation of the SC was effective in evoking rapid eye jumps in monkeys. In 2018, Matsumoto et al. [80] injected an adeno-associated virus type 2 vector (AAV2-CMV-ChR2-EYFP) into the unilateral FEF and found that direct stimulation of a signal from the FEF to the SC can regulate the activity of SC neurons; in addition to modulating SC neuronal activity, optogenetic stimulation of the FEF-SC pathway can also induce saccadic eye movements.

Some researchers have suggested that there may be multiple reasons for the apparently weak effect of optogenetic stimulation, such as differences in activation mechanisms, differences in the neural population being activated, a small number of effectively transfected cells, difficulties in genetic targeting of neurons, and insufficient light delivery. However, many researchers have made great improvements in the effectiveness of optogenetic stimulation. Ebina et al. [81] used adeno-associated viruses (AAVs) with a tetracycline (TET)-induced gene expression system to amplify the neuronal expression of the induced genes; they expressed ChR2 variants (E123T/T159C) in the motor cortex and induced forelimb movements by stimulating larger areas through a cranial window. Watanabe et al. [82] compared the expression of AAV2, AAV5, and AAV-DJ vectors (constructed from DNA reformulations of eight AAV serotypes) and selected the AAV-DJ vector, which has high fluorescence intensity, a large transduction area, and transduced cell count, and high protein expression. The authors expressed ChR2 in the primary motor cortex forelimb region of macaque monkeys and found that optogenetic intracortical microstimulation (oICMS) by homemade photoelectrodes induced significant cortical activity and that oICMS could elicit significant forelimb movements and muscle activity. Rajalingham et al. [83,84] developed a long-term implantable light-emitting diode array that can be used for high-throughput optogenetic interference. In this study, the primary visual cortex of macaque monkeys was silenced through optogenetics, and obvious visual defects were found in the brightness discrimination task, indicating that optical arrays are effective in the brains of macaque monkeys.

Approaches to elective neuronal manipulation through optogenetics can be roughly divided into those that use cell-type-specific gene promoters and those that use projection targeting [73]. Both of these methods are based on the use of viral vectors to transfer the opsin gene into neurons. Due to their safety and ability to effectively transduce postmitotic neurons, AAV and lentivirus vectors have become the standard of optogenetics in macaques [84].

### 3.4. Brain Neural Circuit Research in NHPs

The brain is a dynamic network system. Neurons are intricately connected through synapses, forming the structural basis for the brain to perform complex activities. To fully understand the synaptic connection and information transmission of neurons in different regions, it is necessary to use some tracers to indicate the overall structure of the neural circuit. Early chemical tracers were widely used to study neuronal connections between different brain regions and made great contributions to the understanding of human neurological diseases, such as the anterograde tracers *Phaseolus vulgaris* leucoagglutinin (PHA-L) [85] and biotinylated dextran amine (BDA) [86] as well as the retrograde tracers cholera toxin B subunit (CTb) [87], Fluoro-Gold (FG) [88] and horseradish peroxidase (HRP) [87]. However, the labeling time of these chemical tracers is relatively short, which makes it impossible to achieve multilevel neural network labeling, and no cell type specificity is available [89].

Recombinant viruses have provided a new vision for the study of neural circuits and have become the most commonly used tools. These viral vectors are summarized in Table 1. Recombinant viruses bind recombinant enzymes (e.g., Cre or Flp) or specific promoters to visualize the structure of neural circuits by specifically tracking the cell bodies, projections, and synaptic connections of neurons using viruses that express fluorescent proteins.

#### 3.4.1. Common Viral Vectors in Neural Circuit Research

Viral tracers that are widely used in rodents include AAV [90,91,92], canine adenovirus-2 (CAV-2) [93], herpes simplex virus (HSV) [94], pseudorabies virus (PRV) [95], and rabies virus (RV) [93,96]. Based on the ability of viruses to cross synapses, viral tracers can be classified into two categories: non-transsynaptic and transsynaptic (including trans-multipolar and trans-unipolar). Non-transsynaptic viruses cannot cross synapses to reach other neurons and are restricted to infected neurons, whereas transsynaptic viruses can cross synapses and spread to other synaptically connected neurons. According to the direction of viral transport along the axon, transsynaptic viral tracers can be further classified as anterograde or retrograde. Common virus vectors for neural circuit tracing are shown in Table 1.

#### 3.4.2. Application of Neural Circuit Tracing in the Study of Emotion in NHPs

Emotions, such as happiness, satisfaction, anxiety, and fear, are physiological and psychological states produced by the synthesis of a variety of complex feelings, thoughts, and behaviors. Emotions enrich our lives, but strong and persistent negative emotions affect people’s health and lead to extreme behavior.

##### Current Status of Research and Application of Neural Circuit Tracing of Anxiety in NHPs Models

Anxiety, a highly aroused and negative state, can be triggered by stimuli that do not directly constitute a danger or can be generated internally. In NHP studies, pathological or trait anxiety has been shown to be associated with the dorsal amygdala, including the central nucleus and the dorsal part of the basolateral nucleus [97].

Extensive rodent studies have shown that many amygdala subregions are involved in regulating anxiety-related behaviors [98,99,100]. For example, inhibition of projections from the basolateral amygdala to the ventral hippocampus reduces anxiety-related behaviors, whereas increased activity in these projections enhances anxiety-like behaviors [98]. Inhibition of basolateral amygdala projections into the lateral central nucleus can also increase anxiety-related behaviors [100]. The interaction between the BLA and the bed nucleus of the stria terminalis (BNST) has also been demonstrated to modulate anxiety-like behaviors. The input from the BLA to the anterior dorsal BNST exerts an anxiolytic effect on behavior and physiology. The local inhibition of the anterior dorsal BNST by the oval nucleus of the BNST causes anxiety [101], which is mediated by the projections from the anterior dorsal BNST to the ventral tegmental area (VTA). The ventral BNST regulates anxiety through its innervation of the VTA, with the activity of glutamatergic ventral BNST neurons projecting to the VTA producing anxiety-like behavior and ventral BNST gamma-aminobutyric neurons producing anti-anxiety effects through parallel pathways [102].

Clozapine-N-oxide (CNO) was originally considered an ideal activator of designer receptors exclusively activated by designer drugs (DREADDs), with high affinity and selectivity for DREADDs, and has been widely used in rodent studies to mediate amygdala inhibition to regulate anxiety. Some studies using rAAV5/hSyn-hM4D-mCherry to transfect the amygdala of rhesus monkeys have revealed that CNO reduces the resting-state functional connectivity between the amygdala and the frontal cortex by binding to the receptor DREADD of hM4Di. However, this study did not use DREADD-free mice as a control group. Recent pharmacokinetic evidence shows that CNO has poor brain permeability, but CNO can be reversely metabolized to clozapine. Therefore, some doubt was expressed as to whether the observed effect is due to the intrinsic effect of CNO or reverse metabolism to clozapine [67]. Subsequently, Roseboom et al. [97] used AAV5-hSyn-HA-hM4Di to transfect dorsal amygdala neurons in rhesus monkeys and induced the hM4Di binding receptor DREADD to mediate amygdala inhibition by low-dose [^11^C] clozapine. The study demonstrated that low-dose [^11^C] clozapine activated hM4Di in the dorsal amygdala, producing a significant reduction in anxiety-related behaviors in NHPs.

##### Current Status of Research and Application of Neural Circuit Tracing of Fear in NHPs Models

Fear, a vital emotion for the survival of animals, can be generated when individuals are in danger or when they see their companions in danger [103]. However, the neural mechanisms of fear have been controversial in the fields of psychology and neuroscience.

The amygdala, PFC, and hippocampus have been identified as three key brain regions involved in the learning, regulation, and regression of the animal fear response [104]. The amygdala is a key region in the medial temporal lobe of the brain. It receives input signals from the thalamus and coordinates the response to threat signals by sending output signals to the hypothalamus [105]. The hippocampus is involved in the fading of fear memories and plays a role in the downregulation of amygdala responses to these signals [106]. The medial prefrontal cortex controls the fear response, receiving input from the hippocampus and sending output through projections to the amygdala to regulate fear behavior [107].

Montardy et al. [108] studied the fear response of marmosets to the presence of predators (moving snakes), characterizing the functional connections of the ventromedial part of the hypothalamus through c-Fos immunolabelling and anterograde/retrograde tracing, and found that the defense system of the medial hypothalamus may play an important role in the internal threat state of primates. The neural mechanisms of fear regulation in rodents have been extensively studied but less so in NHPs. Due to the diverse processes of fear acquisition and the complex neural circuits involved, there are still some difficult problems to be solved. Therefore, various animal models and human studies are needed to characterize the cellular and molecular mechanisms behind fear-related diseases.

#### 3.4.3. Application of Neural Circuit Tracing to the Study of Cognition in NHPs

Cognition refers to a complex set of brain functions, ranging from attention, language, and visuospatial processes to learning, memory formation, and execution [109], including perception, thinking, imagination, and speech.

##### Current Status of Research and Application of Neural Circuit Tracing of Perception in NHP Models

Perception is a series of processes of awareness, sensation, and attention directed towards internal and external information. Up to half of the regions in the macaque cortex and approximately one-quarter of the human cortex are involved in the processing of visual sensory information [110]. In primate brains, the visual system consists of two main pathways: the ventral pathway and the dorsal pathway. The ventral pathway projects from the primary visual cortex (V1) through the ventral occipital lobe to the anterior temporal cortex and is responsible for shape and color perception. The dorsal pathway projects from V1 through the dorsal occipital lobe to the posterior parietal cortex and is responsible for spatial processing and visuomotor control [111]. On the exposed surface of the upper parietal cortex of primates, there are also two regions defined by cell structure, namely, the posterior dorsal part of the superior parietal lobule (area PE) and the caudal part of the superior parietal lobule (area PEc), which are also considered associated with cognition. Gamberini et al. [112] injected the retrograde tracer bound to wheat germ agglutinin and horseradish peroxidase in six cynomolgus monkeys and found that the claustrum sent projections to the partially overlapping areas PE and PEc, forming projections to the posterior parietal lobe and the somatosensory, visual and motor cortices. Most of the labeled neurons were in the posterior dorsal part of the claustrum. Area PE receives additional input from the posterior dorsal region of the claustrum, while area PEc receives additional input from the anterior ventral region of the claustrum. The former contains neurons mainly involved in somatosensory processing, while the latter includes somatosensory and visual neurons. The results showed that claustrum projections may help coordinate the activity of an extensive neural circuit involved in sensory and motor processing for movement execution.

In addition, both human and NHP studies have revealed the existence of a third visual pathway on the lateral surface of the brain. The third pathway projects from the early visual cortex to the superior temporal sulcus (STS) through the motor selection area. STS is used to calculate the movement of the moving face and body (for example, expressions, gaze, audio–visual integration, intention, and mood), indicating that the third visual pathway is dedicated to the dynamic aspects of social perception [113].

##### Current Status of Research and Application of Neural Circuit Tracing of Memory in NHPs Models

The hippocampus and PFC are considered of critical importance for cognition. The hippocampus is an important component of the limbic system in the human brain and plays an important role in spatial navigation and the integration of information from short-term to long-term memory [114]. Extensive studies in rodents have shown that the hippocampal pathway originates in the temporal two-thirds of CA1 and the subiculum and terminates in specific layers of the infralimbic (IL) and prelimbic (PL) regions of the medial prefrontal cortex. Some studies have reported that this pathway plays a key role in decision-making, goal-oriented behavior, working memory, and emotional regulation [115], but more validation is needed in NHP research.

The dorsolateral prefrontal cortex (dlPFC) is unique to primates and underlies their unique cognitive abilities. It is located at the core of advanced brain function through the neural circuit connecting the dorsal caudate nucleus (dCD) and the medial thalamus (MDI). Oyama et al. [116] transfected the bilateral PFC with AAV1-hSyn-hM4Di-IRES-AcGFP and the unilateral PFC with AAV2-CMV-mKO in macaque monkeys. They found that silencing of bilateral dlPFC-MDI projections impaired the performance of spatial working memory tasks, whereas silencing unilateral dlPFC-dCD projections only changed preferences in decision-making tasks, suggesting separation of the prefrontal–subcortical pathway in working memory and decision-making. However, the unique mechanism of these prefrontal–subcortical pathways is still unclear, and unraveling these complex brain regions is critical to how the organism processes information and how instructions are executed.

## 4. Outlook

NHPs are of irreplaceable value in the field of cognitive research. In the clinic, brain cognitive-related diseases still have a high mortality rate, and there is no effective treatment. Although many scholars have conducted extensive research in the field of neuroscience and achieved a series of great achievements, some NHP brain disease models cannot effectively simulate the common clinical symptoms and typical pathological markers of the diseases. In the future, more attention should be given to the exploration of NHP disease models.

In addition, electrophysiological, optogenetic, chemogenetic, and other techniques have made positive achievements in the study of brain function in NHPs. Major breakthroughs will be made in the cognitive field when the cognitive control mechanisms of the brain for different tasks are revealed by studying specific brain areas/neurons and identifying neural networks corresponding to behaviors, neural circuits, and causal interactions between neurons. However, there are still some problems in the study of NHPs. For example, the primate brain is two orders of magnitude larger than the mouse brain, which makes it more difficult to research the deep brain in NHPs. Additionally, the behavioral response induced by the regulation of the primate brain is far more complex than that of rodents. We reviewed the methods of constructing animal models of some brain diseases in NHPs and the applications of neuromodulation and imaging techniques in NHPs. We hope that in the future, NHP neuromodulation techniques and imaging methods can be applied to the research of NHP brain diseases, thereby promoting the development and research of these diseases.

## Figures and Tables

**Table 1:** Common viral vectors for the study of neural circuits.

Virus Name	Direction	Ability to Cross Synapses	Genomic Type	Advantages and Disadvantages
AAV9	Anterograde	Non-transsynaptic	ssDNA	Steady expression for several months, strong capacity for infection and transmission
AAV2/9	Anterograde	Non-transsynaptic	ssDNA	High efficiency in long-distance axon tracing
CAV2	Retrograde	Non-transsynaptic	dsDNA	Efficient retrograde transport; low cellular tropism
rAAV2-retro	Retrograde	Non-transsynaptic	ssDNA	Stable transgene expression; efficient retrograde transport
VSV	Anterograde	Polysynaptic	(-) ssRNA	Fast expression, strong signal, high transsynaptic efficiency, but relatively high toxicity
HSV-1-H129	Anterograde	Polysynaptic	dsDNA	Fast expression, strong signal, high transsynaptic efficiency, but relatively high toxicity
HSV-1-H129-ΔTK	Anterograde	Monosynaptic	dsDNA	Fast expression, strong signal, high transsynaptic efficiency, cell-specificity, but relatively high toxicity
AAV1	Anterograde	Monosynaptic	ssDNA	Cell specificity
PRV-152	Retrograde	Polysynaptic	dsDNA	Efficient retrograde transport, but greater toxicity
Wild-type RV	Retrograde	Polysynaptic	(-) ssRNA	Lack of cytopathic effects
EnvA-RVΔG	Retrograde	Monosynaptic	(-) ssRNA	Cell specificity

## Data Availability

Data available in a publicly accessible repository.
